# Rehabilitation Outcomes in Multiple Sclerosis Patients on Ocrelizumab Diagnosed With West Nile Virus Encephalitis

**DOI:** 10.7759/cureus.57063

**Published:** 2024-03-27

**Authors:** Taylor R Johnson, Stephanie Gandelman, Lauren R Serafin, Jeremy Y Charles, Dina Jacobs

**Affiliations:** 1 Physical Medicine and Rehabilitation, Hospital of the University of Pennsylvania, Philadelphia, USA; 2 Neurology, Westchester Medical Center, Valhalla, USA; 3 Neurology, Hospital of the University of Pennsylvania, Philadelphia, USA

**Keywords:** west nile virus encephalopathy, brain injury rehab, outcome of inpatient rehabilitation, west nile neuro-invasive disease, ocrelizumab, multiple sclerosis, physical medicine and rehabilitation (pm&r)

## Abstract

Multiple sclerosis (MS) has a global prevalence exceeding two million people and is a leading cause of non-traumatic physical disability. MS can be treated with ocrelizumab, an anti-CD20 monoclonal antibody. West Nile virus (WNV) is the most common cause of mosquito-borne viral encephalitis in North America. It can lead to neuroinvasive WNV disease (WNND) affecting the brain and peripheral nervous system, especially in immunocompromised patients, such as those being treated with ocrelizumab for MS. WNND is exceedingly rare and reported in less than 1% of cases of WNV. It has been established that inpatient rehabilitation improves functional outcomes in patients with MS and those with WNND. However, the inpatient rehabilitation outcomes in patients diagnosed with both WNND and MS have not been reported.

In this study, we aimed to examine the rehabilitation outcomes of MS patients on ocrelizumab diagnosed with WNND. We performed a retrospective chart review of patients with MS treated with ocrelizumab, who were diagnosed with WNND and admitted to a single facility. Rehabilitation outcomes were assessed using functional independence measure (FIM) scores on admission and discharge. Three patients met the inclusion criteria; two in acute rehab, and one in the long-term acute care hospital (LTACH). Both patients admitted to acute inpatient rehabilitation showed an improvement in FIM scores from admission to discharge, one patient from 9 to 16 and the other from 14 to 54. However, the patient admitted to the LTACH had no improvement in FIM score from admission to discharge. Patients admitted to acute rehab were ultimately discharged home, while the patient admitted to the LTACH required discharge to a subacute rehabilitation facility.

Based on our findings, intense and prolonged comprehensive inpatient rehabilitation is associated with improved functional outcomes and increased likelihood of discharge to home in this population suffering from both central and peripheral nervous system involvement due to MS and WNND.

## Introduction

Multiple sclerosis (MS) is a chronic, autoimmune, demyelinating disorder of the central nervous system (CNS) [1-6] with a prevalence exceeding two million people worldwide [3]. It is a leading cause of non-traumatic physical disability in young adults [3,6]. In recent years, the classic paradigm of MS as a primarily T-cell-driven disease has been challenged by evidence that revealed B-cells playing a larger role in pathogenesis than previously understood [1,2,6], leading to the development of selective B-cell depleting disease-modifying therapies (DMTs) that specifically target CD20. These new therapies have proven to be highly efficacious in reducing MS disease activity. One of these agents, ocrelizumab, was approved by the FDA in 2017 [2] and has had a rapid uptake in the MS community due to its infrequent administration, high efficacy, and overall limited toxicity and side effect profile. However, though it is generally well-tolerated, ocrelizumab is associated with an increased frequency of mild infections due to its immunomodulatory properties [1,2,5,6]. Notably, most infections related to ocrelizumab administration are mild upper respiratory infections or urinary tract infections (UTIs) and reports of serious infections have been rare [5,6].

West Nile virus (WNV) is the most common cause of mosquito-borne viral encephalitis in North America [7,8]. Neuroinvasive WNV disease (WNND) is seen in <1% of cases and can manifest as encephalitis, meningitis, myelitis, flaccid paralysis, altered consciousness, and seizures [8,9]. It is well-documented that the most severe infections with WNV occur in the elderly and immunocompromised patients [9,10]. WNND can lead to devastating cognitive and functional morbidity, and even death [11]. Humoral immunity plays a crucial role in neutralizing WNV infection [10], and immunosuppressive therapy is associated with a worse prognosis following WNV infection [11]. A recent report describes four cases of WNND in patients with MS receiving ocrelizumab without other risk factors [12]. The diagnosis of WNV infection is difficult in patients who have received prolonged treatment with ocrelizumab, due to the challenges of isolating serum WNV antibodies. WNV-positive antibodies were quickly isolated in the serum of a patient who had received the fewest ocrelizumab infusions. These findings fit with extended B-cell depletion, where the formation of new antibodies is attenuated.

Patients diagnosed with MS and WNND may present with severe neurologic deficits due to the involvement of both the central and peripheral nervous systems. There is mounting evidence that rehabilitation intervention improves outcomes in functional mobility and activities of daily living (ADLs) as measured by admission and discharge functional independence measure (FIM) scores in patients with WNND [8,13]. Similarly, the benefit of inpatient rehabilitation for patients with functional, sensory, visual, and cognitive deficits due to MS has been well described in the literature. A systematic review of Cochrane review articles by Amatya et al. in 2019 concluded that there is moderate-quality evidence that physical therapeutic modalities improved functional outcomes, reduced impairment, and improved participation in patients with MS [14]. Commonly cited benefits following inpatient rehabilitation include decreased fatigue, improved walking speed, and improved quality of life in MS patients [14,15]. Mobility is the most frequently reported area of improvement for patients with MS following a course of inpatient rehabilitation; however, baseline mobility is the most important predictive factor as to whether a patient will attain mobility goals [16-19,15]. Shorter disease course, longer rehabilitation length of stay (LOS), female sex, and relapsing subtype may be associated with better rehabilitation outcomes [20].

Although inpatient rehabilitation offers the ideal setting to maximize recovery in these patients, to our knowledge, the inpatient rehabilitation outcomes in patients with MS diagnosed with WNND have not been reported. Acute rehab in such individuals can be quite challenging as patients with MS may have a pre-existing disability that is compounded by WNND, a condition that impacts both cognitive and physical function and has an exceedingly slow recovery period. Prolonged and intense rehab is necessary to allow for meaningful functional recovery that may allow patients to ultimately return home to live independently.

We present a case series of three individuals with MS treated with ocrelizumab who developed WNND and subsequently received inpatient rehabilitation. We performed a retrospective chart review per the Institutional Review Board guidelines. The inclusion criteria were as follows: patients with MS treated with ocrelizumab, who were diagnosed with WNND and admitted to a single facility, housing both an acute inpatient rehabilitation hospital and a long-term acute care hospital (LTACH), from September to November 2022. The primary aim of this retrospective case series is to analyze the rehabilitation outcomes in MS patients on ocrelizumab diagnosed with varying severities of WNND. Our secondary aim is to add to the current body of literature on rehabilitation by demonstrating the utility of acute inpatient rehabilitation for patients with both MS and WNND.

## Case presentation

The baseline functional status and neurologic deficits of all subjects are presented in Table 1. FIM at the time of admission and discharge for cases one, two, and three are presented in subsequent tables. Of note, an abbreviated version of the comprehensive FIM instrument was utilized, wherein the FIM scale ranged from 8 to 64, rather than 18 to 126. Some FIM categories were not attempted due to medical conditions or the safety of the patient and/or medical staff. For calculation purposes, these were counted as “dependent” or an FIM score of “1” was assigned.

**Table 1:** Baseline functional status and neurologic deficits in all subjects ADLs: activities of daily living

	Functional mobility	ADLs	Neurologic deficits
Case 1	Modified independent; used scooter in community	Required assistance	Right > left lower extremity weakness
Case 2	Independent	Independent	Mild numbness/tingling in bilateral feet
Case 3	Independent	Independent	Left > right hand numbness, spasms

Case one

A female in her early 60s with a past medical history of MS (diagnosed in 1997 and well-controlled on ocrelizumab since 2017), gastroesophageal reflux disease, and hypothyroidism presented to an unaffiliated acute care hospital in August 2022 with fevers, weakness, and confusion. Lumbar puncture (LP) revealed elevated white blood cell (WBC) count with >90% lymphocytes along with elevated protein. Cerebrospinal fluid (CSF) cytology, herpes simplex virus (HSV), and varicella zoster virus (VZV) polymerase chain reaction (PCR) were negative. Serum infectious and inflammatory tests, including WNV IgG and IgM, were negative. The patient developed subacute extremity weakness and encephalopathy, and the exam was notable for paraplegia and absent reflexes. 

**Figure 1:**
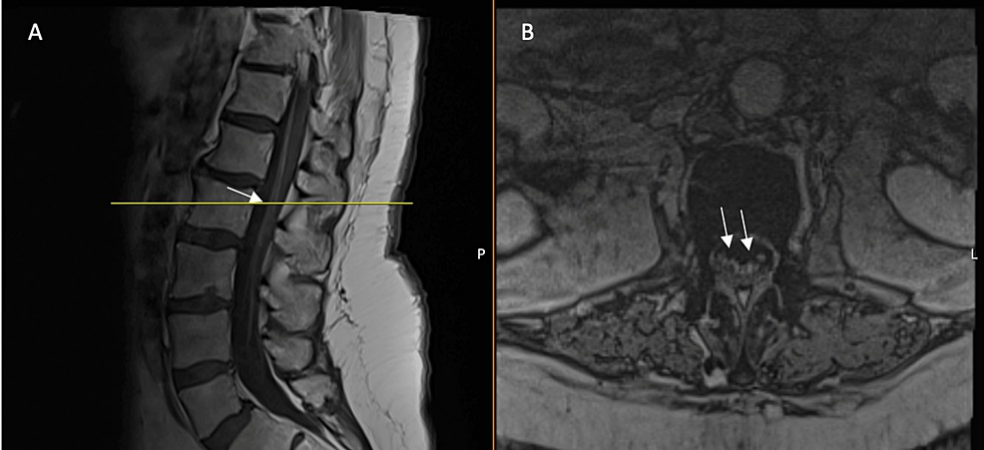
Contrast enhancement of the cauda equina nerve roots (arrows) on postcontrast T1-weighted lumbar spine MRI Sagittal (A) and axial (B) views. The horizontal line indicates the lumbar vertebral level MRI: magnetic resonance imaging

She was transferred to our affiliate acute care hospital. On arrival, a spine MRI revealed avid cauda equina nerve root enhancement (Figure 1). Electromyography (EMG) and nerve conduction studies (NCS) obtained one week apart in August revealed progressive findings suggestive of motor axonal polyradiculopathy without any evidence of demyelination. The patient completed a five-day course of intravenous immunoglobulin (IVIG) with minimal improvement. Repeat CSF cytology and HSV PCR were negative. CSF metagenomics eventually returned positive for WNV. She was diagnosed with WNND. Her hospital course was complicated by ventilator-dependent respiratory failure, multiple bouts of ventilator-acquired pneumonia, and mucus plugging, all stemming from an inability to clear secretions due to neuromuscular weakness. She required tracheostomy and percutaneous gastrostomy (PEG) tube placement. The PM&R team was consulted to evaluate the patient for acute inpatient rehabilitation and they recommended initially transitioning to a long-term acute care hospital for ventilator weaning.

In October 2022, the patient was transferred to an LTACH within the same facility as our inpatient rehabilitation hospital. She received therapeutic interventions, though at a decreased frequency as compared to an acute rehab setting. She made minimal progress with therapies over her 43-day LOS (Table 2). The PM&R consult team continued to follow and evaluate the patient in the hope of transferring her to the acute rehabilitation floor; however, it was deemed more suitable to transfer her to subacute rehabilitation due to her failure to show progress with therapies at the LTACH. At discharge, She remained dependent for functional mobility and ADLs and required mechanical lift. She had no movement of the lower extremities. She could not be safely decannulated and could not tolerate a passy muir speaking valve due to severe neuromuscular weakness. She tolerated a diet of puree and nectar-thickened liquids.

**Table 2:** FIM at the time of admission and discharge for case one FIM: functional independence measure

Category	Admission FIM	Discharge FIM
Toileting	Dependent (1)	Dependent (1)
Shower/bathing	Dependent (1)	Dependent (1)
Upper body dressing	Dependent (1)	Dependent (1)
Lower body dressing	Dependent (1)	Dependent (1)
Rolling side to side	Dependent (1)	Dependent (1)
Sit to stand	Dependent (1)	Dependent (1)
Walking 50 ft	Not attempted due to medical condition or safety	Not attempted due to medical condition or safety
Locomotion	Not attempted due to medical condition or safety	Not attempted due to medical condition or safety

Case two

**Figure 2:**
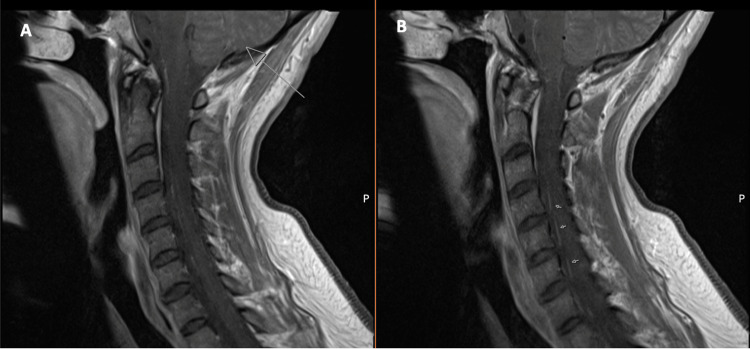
Sagittal postcontrast T1-weighted cervical spine MRI showing enhancement (arrows) of cerebellar folia (A) and multiple ventral nerve roots (B) MRI: magnetic resonance imaging

A female in her early 40s with a past medical history of MS (diagnosed in 2017, well-controlled on ocrelizumab since 2017), basal cell carcinoma of the skin, and depression was admitted to our affiliate acute care hospital in September 2022 with complaints of fevers, cough, vomiting, and intractable headache shortly after a hiking trip. LP revealed an elevated WBC count, a normal glucose level, and minimally elevated protein consistent with a viral infection. Given her known history of anti-CD20 therapy, she received empiric bacterial meningitis antibiotic and steroid coverage. Extensive CSF and serum infectious workup, including bacterial, viral, and fungal studies, was unremarkable. Given recent exposures, the workup for tick-borne illnesses was pursued, including serum histoplasmosis, Leptospira, anaplasia, Ehrlichia, VZV, and Powassan studies. All initial infectious workups were negative and the medication regimen was de-escalated. After dexamethasone was stopped, the patient experienced an acute worsening in mental status, fatigue, and word-finding difficulties. MRI brain revealed leptomeningeal enhancement in the cerebellum and spine MRI revealed enhancement of multiple ventral nerve roots (Figure 2). On hospital day four, WNV Ab IgM was positive in the CSF, establishing the diagnosis of WNND. The remaining antibiotics were stopped, and she completed a five-day course of IVIG. Her mental status and strength improved minimally. The PM&R consult team recommended transfer to acute inpatient rehabilitation in September 2022.

Her rehab course was notable for ongoing confusion and urinary retention requiring an indwelling Foley catheter. The patient made minimal functional progress after 14 days of intensive therapies at our rehabilitation hospital (Table 3). She was then transferred to another inpatient rehabilitation facility where her functional progress was also minimal over an additional 32-day LOS (Table 4). The patient's comprehension, expression, memory, insight, and reasoning improved. After nearly two months in acute rehab, she was discharged home. The patient required a hospital bed, portable lift, commode, and tilt-in-space wheelchair as well as home physical and occupational therapy (PT/OT) and speech two to three times per week for one hour each, nursing care once to twice per week for 20-30 minutes, and a home health aide two to three times per week for one hour. The Foley catheter had been successfully removed before discharge. The patient was tolerating a regular diet and thin liquids at the time of discharge.

**Table 3:** FIM at the time of admission and at the time of transfer to another inpatient rehab facility for case two FIM: functional independence measure

Category	Admission FIM	Transfer FIM
Toileting	Dependent (1)	Dependent (1)
Shower/bathe	Dependent (1)	Substantial/max assist (2)
Upper body dressing	Dependent (1)	Partial/moderate assist (3)
Lower body dressing	Dependent (1)	Dependent (1)
Rolling side to side	Substantial/max assist (2)	Substantial/max assist (2)
Sit to stand	Not attempted due to medical condition or safety	Dependent (1)
Walking 50 ft	Not attempted due to medical condition or safety	Dependent (1)
Locomotion	Dependent (1)	Dependent (1)

**Table 4:** FIM at the time of transfer from our inpatient rehab and at discharge from another inpatient rehab for case two FIM: functional independence measure

Category	Transfer FIM	Discharge FIM
Toileting	Dependent (1)	Max assist (2)
Shower/bathe	Substantial/max assist (2)	Max assist (2)
Upper body dressing	Partial/moderate assist (3)	Partial/moderate assist (3)
Lower body dressing	Dependent (1)	Substantial/max assist (2)
Rolling side to side	Substantial/max assist (2)	Partial/moderate assist (3)
Sit to stand	Dependent (1)	Max assist (2)
Walking 50 ft	Not attempted due to medical condition or safety	Not attempted due to medical condition or safety
Locomotion	Not attempted due to medical condition or safety	Not attempted due to medical condition or safety

Case three

**Figure 3:**
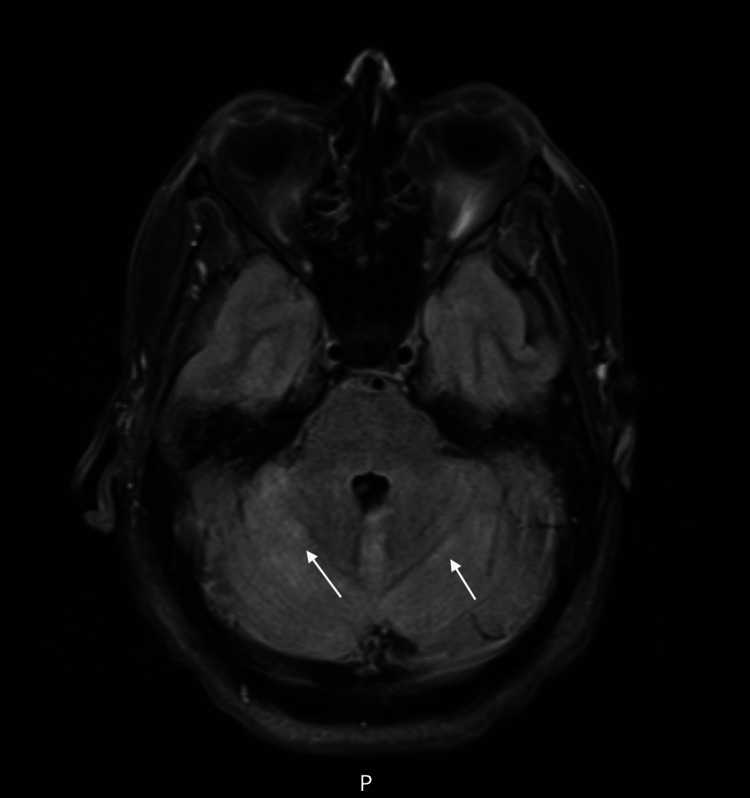
Hyperintense signals in the cerebellum (arrows) on FLAIR MRI of the brain, axial view FLAIR: Fluid-attenuated inversion recovery; MRI: magnetic resonance imaging

A female in her early 40s with a past medical history of MS (diagnosed in 2021, on ocrelizumab since April 2022) and thyroid cancer treated with thyroidectomy (2021) was admitted to an unaffiliated acute care hospital in October 2022 with fevers, headache, and confusion and was found to have a lymphocytic pleocytosis (CSF with WBC of 104, 92% lymphocytes) concerning for encephalitis. The patient had been on a recent hiking trip. She was empirically treated for bacterial meningitis. The patient's mental status was noted to wax and wane, raising concern for seizures; she was transferred to our affiliate acute care hospital for EEG monitoring, which revealed non-convulsive status epilepticus, ultimately controlled on multiple antiepileptic agents. She was intubated and required prolonged weaning off from sedation. MRI brain revealed new fluid-attenuated inversion recovery (FLAIR) hyperintensities in the cerebellum felt to reflect acute cerebellitis (Figure 3). Repeat CSF testing for HSV, VZV, and enterovirus, and serum testing for HIV, Babesia, Ehrlichia, Anaplasma, and Rickettsia IgG/IgM were negative. She received empiric ceftriaxone and doxycycline until Lyme and Rickettsia testing returned negative. Serum studies eventually returned positive WNV IgG and IgM. She was diagnosed with WNND and treated with IVIG with minimal improvement in symptoms. The PM&R consult team recommended transfer to acute inpatient rehabilitation in October 2022.

While at rehab, the patient initially had urinary retention and required clean intermittent catheterization (CIC) every six hours. She required scheduled CIC until day five of admission when she began voiding spontaneously with low post-void residuals. Home baclofen was resumed for baseline muscle spasms of bilateral hands. Levetiracetam was continued for seizure management and there was no further concern for seizure activity. The patient made excellent progress with functional mobility and ADLs (Table 5). After a 20-day LOS, the patient was discharged home with a rolling walker, commode, tub bench, and adaptive dressing equipment. She received at-home PT/OT. She demonstrated mild, higher-level cognitive-linguistic impairments in the areas of memory. The patient scored above the normal range on the Cognitive Linguistic Quick Test. She was tolerating a regular diet and thin liquids without signs of aspiration on discharge from rehab.

**Table 5:** FIM at the time of transfer from other inpatient rehab and at discharge for case three FIM: functional independence measure

Category	Admission FIM	Discharge FIM
Toileting	Dependent (1)	Independent (7)
Shower/bathe	Dependent (1)	Independent (7)
Upper body dressing	Dependent (1)	Independent (7)
Lower body dressing	Dependent (1)	Independent (7)
Rolling side to side	Supervision/touching assist (5)	Independent (7)
Sit to stand	Partial/moderate assist (3)	Independent (7)
Walking 50 ft	Not attempted due to medical condition or safety	Independent (7)
Locomotion	Not attempted due to medical condition or safety	Supervision/touching assist (5)

LOS, average hours of comprehensive therapies per week, exact change in FIM scores, and discharge disposition for all patients are reported in Table 6. 

**Table 6:** LOS, hours of therapies per week (average), change in total FIM score (exact), and discharge disposition for all subjects LOS: length of stay; FIM: functional independence measure; SAR: subacute rehabilitation

	LOS in days	Hours of therapy per week (avg.)	Change in FIM score	Discharge disposition
Case 1	43	5	0	SAR
Case 2	46	15	+7	Home
Case 3	20	15	+40	Home

## Discussion

This case series discusses three individuals with MS on ocrelizumab who developed neuroinvasive WNV infection and required admission to acute inpatient rehabilitation, where they received comprehensive therapeutic interventions for an average of 15 hours per week, or to an LTACH, where the primary goal of the admission revolved around the management of ongoing acute medical needs and therapy was delivered on an as-tolerated basis.

All patients included in the series received the full course of therapeutic interventions routinely offered within an inpatient rehabilitation setting. These interventions included physical therapy, occupational therapy, speech-language pathology (SLP), and neuropsychology if needed. The degree to which each therapeutic discipline was required varied based on the severity of WNND. Each patient participated in comprehensive therapy programs designed to meet their individual needs. The patients described in cases one and two required more SLP intervention given their significant encephalopathy and difficulties with oral feeding. After receiving therapy at the acute rehab level, the patient in case two was eventually able to tolerate a regular diet with thin liquids. In contrast, the patient in case one continued with a modified diet at the time of discharge from LTACH. After presenting with status epilepticus and experiencing waxing and waning mental status changes during the acute care admission, the patient presented in case three had an almost complete recovery of her mental status baseline while admitted to inpatient rehab. 

LOS varied among the patients studied. Two of the three patients were able to return home. The one patient who was admitted to the LTACH was discharged to a skilled nursing facility due to the burden of her care needs. Both patients who were discharged home received intensive therapies at acute inpatient rehabilitation. A benefit of acute inpatient rehabilitation is the availability of caregivers to offer hands-on caregiver training alongside the therapists who have been treating the patient throughout their rehab admission. This may have played a significant role in allowing the patient in case two to be discharged home despite still requiring maximal assistance for ADLs and functional mobility at discharge.

An interesting finding in this case series was that the patients with a longer disease duration of MS and a longer duration of treatment with ocrelizumab had worse rehabilitation outcomes. The most severe case of WNND was seen in the patient in case one, who was diagnosed with relapsing-remitting MS (RRMS) in 1997 and had been treated with ocrelizumab for five years before WNV infection. The next most severe case of WNND was observed in the patient presented in case two, who was diagnosed with RRMS in 2017 and had also been treated with ocrelizumab for five years before WNV infection. The patient with the least severe neuroinvasive disease was the one presented in case three, who was diagnosed with RRMS in 2021 and had just begun treatment with ocrelizumab six months before WNV infection.

Additionally, we found that patients with EMG or imaging findings consistent with WNV polyradiculopathy had worse rehabilitation outcomes. The patient in case three had relatively benign imaging and was ambulating at the supervision level at the time of discharge. The patient in case two had leptomeningeal enhancement on brain MRI and enhancement of multiple ventral nerve roots on cervical spine MRI. At discharge from inpatient rehab, this patient required maximal assistance for ambulation. Six months later, the patient had a near-complete resolution of WNV-related neuroinflammation and was able to ambulate short distances with a rolling walker in a modified independent manner. In contrast, the patient in case one, who had EMG findings consistent with motor axonal polyradiculopathy, was dependent for ambulation at discharge from rehab and remained unable to ambulate several months after discharge. 

This study adds to the existing body of literature by demonstrating the benefits of acute inpatient rehabilitation in a unique population of patients; however, it has some limitations. Given the rarity of the cases examined, the sample size was small, and included only females, limiting the generalizability of our findings. Additionally, the abbreviated version of the FIM instrument used in this study to measure functional outcomes did not assign scores in areas such as bowel and bladder control, communication, and cognition. Future studies could consider employing the complete FIM instrument, or an alternative validated measure, to report functional outcomes. Finally, many studies on the rehabilitation outcomes of patients with MS include assessments of walking speed and patient-reported quality-of-life surveys, but this study did not. Future studies looking at rehabilitation outcomes in patients with MS may consider including these measures as it would be helpful for comparison.

## Conclusions

The benefits of acute inpatient rehabilitation, specifically brain injury rehabilitation, for the functional recovery of patients with acute MS flares and those with neuroinvasive disease due to WNV infection, have been previously reported. This study found that the same was true for a rare series of patients with both diagnoses. Our findings revealed that longer MS disease duration, longer duration of treatment with ocrelizumab, increased severity of WNND, presence of non-MS-related neuro-inflammation on MRI, peripheral nervous system involvement on EMG, and decreased baseline mobility were each associated with worse functional outcomes following inpatient rehabilitation. Further studies involving larger sample sizes are necessary to better understand these relationships. Additionally, we found that in patients with MS, evidence of WNV-associated inflammation on MRI may actually improve over time and be associated with reversible disability.

Overall, this study demonstrated that comprehensive inpatient rehabilitation is associated with improved functional outcomes and increased likelihood of discharge to home in this unique patient population suffering from both central and peripheral nervous system involvement due to MS and WNND.

## References

[1] Lamb YN (2022). Ocrelizumab: a review in multiple sclerosis. Drugs.

[2] Florou D, Katsara M, Feehan J, Dardiotis E, Apostolopoulos V (2020). Anti-CD20 agents for multiple sclerosis: spotlight on ocrelizumab and ofatumumab. Brain Sci.

[3] Jalkh G, Abi Nahed R, Macaron G, Rensel M (2020). Safety of newer disease-modifying therapies in multiple sclerosis. Vaccines (Basel).

[4] Margoni M, Preziosa P, Tortorella P, Filippi M, Rocca MA (2022). Does ocrelizumab limit multiple sclerosis progression? Current evidence from clinical, MRI, and fluid biomarkers. Neurotherapeutics.

[5] Hauser SL, Kappos L, Montalban X (2021). Safety of ocrelizumab in patients with relapsing and primary progressive multiple sclerosis. Neurology.

[6] Kanatas P, Stouras I, Stefanis L, Stathopoulos P (2023). B-cell-directed therapies: a new era in multiple sclerosis treatment. Can J Neurol Sci.

[7] Nazneen F, Bai F (2020). The roles of Osteopontin in the pathogenesis of West Nile encephalitis. Vaccines (Basel).

[8] Patel K, Greenwald BD, Sabini RC (2021). Rehabilitation outcomes in subjects with West Nile neuro-invasive disease. Brain Sci.

[9] Leis AA, Stokic DS (2012). Neuromuscular manifestations of West Nile virus infection. Front Neurol.

[10] Diamond MS, Sitati EM, Friend LD, Higgs S, Shrestha B, Engle M (2003). A critical role for induced IgM in the protection against West Nile virus infection. J Exp Med.

[11] Owens M, Choe L, Rivera JE, Avila JD (2020). West Nile virus neuroinvasive disease associated with rituximab therapy. J Neurovirol.

[12] Thebault S, Gandelman S, Lane C (2023). Severe neuroinvasive West Nile virus in association with anti-CD20 monotherapy for multiple sclerosis. Neurol Neuroimmunol Neuroinflamm.

[13] Rao N, Char D, Gnatz S (2005). Rehabilitation outcomes of 5 patients with severe West Nile virus infection: a case series. Arch Phys Med Rehabil.

[14] Amatya B, Khan F, Galea M (2019). Rehabilitation for people with multiple sclerosis: an overview of Cochrane Reviews. Cochrane Database Syst Rev.

[15] Grasso MG, Pace L, Troisi E, Tonini A, Paolucci S (2009). Prognostic factors in multiple sclerosis rehabilitation. Eur J Phys Rehabil Med.

[16] Gaber TA, Oo WW, Gautam V, Smith L (2012). Outcomes of inpatient rehabilitation of patients with multiple sclerosis. NeuroRehabilitation.

[17] Salhofer-Polanyi S, Windt J, Sumper H (2013). Benefits of inpatient multidisciplinary rehabilitation in multiple sclerosis. NeuroRehabilitation.

[18] Hvid LG, Gaemelke T, Dalgas U (2021). Personalised inpatient multidisciplinary rehabilitation elicits clinically relevant improvements in physical function in patients with multiple sclerosis - the Danish MS Hospitals Rehabilitation Study. Mult Scler J Exp Transl Clin.

[19] Francabandera FL, Holland NJ, Wiesel-Levison P, Scheinberg LC (1988). Multiple sclerosis rehabilitation: inpatient vs. outpatient. Rehabil Nurs.

[20] Groppo E, Signori A, Sormani MP, Grosso C, Mantia L, Cattaneo D, Rovaris M (2019). Predictors of hospital-based multidisciplinary rehabilitation effects in persons with multiple sclerosis: a large-scale, single-centre study. Mult Scler J Exp Transl Clin.

